# Neural correlates of decision making related to information security: Self-control and moral potency

**DOI:** 10.1371/journal.pone.0221808

**Published:** 2019-09-04

**Authors:** Robert West, Emily Budde, Qing Hu

**Affiliations:** 1 Department of Psychology and Neuroscience, DePauw University, Greencastle, Indiana, United States of America; 2 Paul H. Cook Department of Information Systems and Statistics, Baruch College, The City University of New York, New York, New York, United States of America; Middlesex University, UNITED KINGDOM

## Abstract

Security breaches of digital information represent a significant threat to the wellbeing of individuals, corporations, and governments in the digital era. Roughly 50% of breaches of information security result from the actions of individuals inside organizations (i.e., insider threat), and some evidence indicates that common deterrence programs may not lessen the insiders’ intention to violate information security. This had led researchers to investigate contextual and individual difference variables that influence the intention to violate information security policies. The current research builds upon previous studies and explores the relationship between individual differences in self-control and moral potency and the neural correlates of decision making in the context of information security. The behavioral data revealed that individuals were sensitive to the severity of a violation of information security, and that the measures of self-control and moral potency were reliable indicators of the underlying constructs. The ERP data provided a partial replication of previous research, revealing differences in neural activity for scenarios describing security violations relative to control stimuli over the occipital, medial and lateral frontal, and central regions of the scalp. Brain-behavior analyses showed that higher moral potency was associated with a decrease in neural activity, while higher self-control was associated with an increase in neural activity; and that moral potency and self-control tended to have independent influences on neural recruitment related to considering violations of information security. These findings lead to the suggestion that enhancing moral potency and self-control could represent independent pathways to guarding against insider threat.

## Introduction

Breaches of information security increasingly represent a substantial threat to the wellbeing of private citizens, corporations, and government entities in the digital global ecosystem. Cybersecurity Ventures [1] estimates that in 2015 the costs of cybercrime to the world economy was $3 trillion. Based upon this estimate, cybercrime would have represented the world’s fifth largest economy in 2016 [2]. In addition to the financial costs, breaches of information security may lower consumer confidence [3] and shape national politics and international relations [4]. Given the ubiquity of insider threats to information security (i.e., roughly 50% of security breaches have a substantial insider component [5]), many organizations have relied on deterrence programs as a primary component of security defense. However, evidence indicates that sanctions embodied in deterrence programs may not be a significant predictor of insiders’ intention to violate information security policies [6]. This finding has led researchers to explore contextual and individual difference variables that may be more effective in predicting the insiders’ intention to compromise information security. As an example, drawing on theories of criminology, scholars have found that lower self-control is associated with higher levels of the intention to violate information security policies [6], while higher moral belief is associated with lower levels of the intention to violate information security policies [7]. However, these findings are based on survey data that do not reveal the mechanisms underlying decision making that gives rise to these relationships. The current study extends upon prior research by using event-related brain potentials (ERPs) to examine the association between self-control and moral potency, and the behavioral and neural correlates of decision making in the context of information security.

Scholars within the information systems community have advocated for the utility of incorporating neuromonitoring measures (e.g., functional magnetic resonance imaging (fMRI) or ERPs) into studies examining the processes underpinning decision making within this field [8, 9, 10]. As a testament to the value of neuromonitoring measures, the inclusion of these methods in information systems research has yielded significant insight into the neurocognitive mechanisms that contribute to the ineffectiveness of security warnings related to malware [11], and the neural basis of methods that may serve to enhance the effectiveness of such warnings [12]. Also, Hu et al. [13] used ERPs to examine both the neural correlates of decision making related to information security, and to identify the locus of the influence of self-control on the neural correlates of decision making within this domain.

### Information security paradigm for ERP research

Hu et al. [13] developed the information security paradigm (ISP) to examine the neural correlates of decision making related to information security using ERPs. In this task individuals are first given some background information about Josh, a hypothetical IT specialist who is under pressure at work and experiencing some financial strain. In the task, individuals are asked to answer a series of prompts as if they were Josh. For each trial, individuals first read a scenario that is one to two sentences in length and then advance to a one sentence prompt describing an action that Josh could take based upon the scenario. As implemented in the current study, the task includes three types of scenarios (i.e., control, minor or major violation of information security policy). Control scenarios do not include an ethical consideration, while minor and major violation scenarios include ethical dilemmas that vary in severity. In the task, response choice, response time, and ERPs are locked to the onset of the choice prompt.

With this paradigm, Hu et al. [13] studied ERPs that distinguished control scenarios from minor and major violation scenarios, and minor violation scenarios from major violation scenarios. Differences in the ERPs elicited by scenarios including an ethical consideration relative to control scenarios emerged around 200 ms after presentation of the decision prompt and were then observed for up to 1800 ms after presentation of the prompt. Over the posterior region of the scalp, the effects of a violation on the ERPs reflected both transient activity between 200–500 ms after presentation of the prompt, and more sustained ERP activity that extend over much of the 2000 ms epoch that was analyzed. Distributed source analysis localized the early transient ERP activity to the right occipital-temporal region and the right anterior frontal/frontal-polar regions of the cortex. Following the early transient posterior activity, there was longer lasting sustained ERP activity over the medial and lateral frontal regions of the scalp. This sustained activity was localized to the anterior frontal, lateral frontal, and anterior temporal cortex.

Hu et al. [13] demonstrated that individual differences in self-control were associated with an attenuation of early transient and later sustained ERP activity that distinguished major violation scenarios from control scenarios; and differential sustained ERP activity that distinguished minor violation scenarios from control scenarios over the lateral frontal region. One limitation of the Hu et al. study was the use of a composite variable of self-control that collapsed across the six subscales represented within the measure, making it impossible to determine whether various aspects of self-control are differentially related to ERP activity elicited during decision making. Therefore, in the current study, we examine the association between ERP activity related to information security decision making and each of the six dimensions of self-control.

### Self-control and moral potency

The idea that variation in self-control represents a significant predictor of criminal behavior in general [14] and violations of information security [15] more specifically is well supported by the extant literature. Extensive work examining the predictors of criminal and antisocial behavior reveals the poor self-control may represents one of the strongest predictors of criminal behavior [14]. Additionally, experimental work conducted within the ego depletion framework reveals that the temporary depletion of self-control is associated with an increase in dishonest or unethical behavior [16]. The temporary disruption of self-control is associated with both an increase in the frequency of cheating and an increase in the likelihood that individuals will select an environmental context that affords a greater opportunity to act dishonestly [17]. In the broader context of the current study, there is also some indication that the effect of self-control depletion on cheating behavior may arise from a reduction moral awareness [16].

Within the information security domain, low self-control is related to higher levels of software [18] and digital piracy [19] in college students, hacking behavior in young adults [15], and higher intentions to commit violations of information security in corporate employees [6]. Some evidence indicates that there may be differences in the effects of subcomponents of self-control on unethical behavior related to information security. For instance, risk-taking is related to digital piracy [19] and hacking for either fun or revenge [15], while the ability to regulation emotion (i.e., tempe) is more strongly related to exploratory hacking than exploitative hacking [15]. The effect of self-control on the intention to violate information security is related to an individual's perception of both the extrinsic and intrinsic benefits that may be derived from the unethical act [6], indicating that variation in the function or sensitivity of the reward system in individuals with low self-control could contribute to violations of information security.

The importance of high moral character within the business world has been widely appreciated and represents a fundamental tenet of both moral potency theory [20] and moral foundations theory [21]. In the decision making literature there is clear evidence that priming moral beliefs related to fairness, correctness, and generousity has a strong effect on cooperation and prosocial behavior that may override the influence on concepts related to the equitable and efficient distribution of resources in a variety of tasks [22,23]. In considering the potential intersection of self-control and moral belief, there is evidence that both coorperation and ethical behavior require the deployment of controlled deliberative processes [24], and that the availability of controlled processes may interact with individual differences in moral identity to predict the frequency of unethical behavior [16].

In the context of information systems, moral belief in the appropriateness of an action is known to be a reliable predictor of violations of information security [25,26]. As an example, moral belief represents a moderate to strong unique predictor of both exploratory and exploitative hacking behavior and the strength of this effect is reasonably consistent regardless of the motivation for the unethical behavior [15]. For instance, the strength of the effect of moral belief on hacking for fun is similar to the strength of the effect of moral belief on hacking for profit [15]. Moral beliefs may also be related to the intention to commit violations of information security in corporate professionals, and this effect may be related to the perceived risks [6] and benefits [27] associated with the unethical act. In the current study, we sought to examine the association between the behavioral and neural correlates of decision making related to information security and general moral beliefs as measured in the Moral Potency Questionnaire [20,28] in order to consider the influence of individual differences in moral beliefs beyond the context of the unethical actions considered in previous research that were specifically related to information security [15,26].

### Functional neuroanatomy of ethical decision making

There has been limited research examining the functional neuroanatomy of ethical decision making related to information security [13]; in contrast, significant progress has been made in characterizing the neural networks that underpin ethical or moral reasoning more generally [29]. In synthesizing this literature, Greene [29] notes that moral reasoning emerges from the interaction of a distributed neural network where none of the elements of the network are uniquely dedicated to moral reasoning. Studies incorporating fMRI and typical individuals reveal that moral reasoning is consistently associated with the recruitment of structures within the frontal cortex including the anterior cingulate cortex, middle frontal gyrus, medial frontal gyrus, and ventromedial prefrontal cortex [30,31,32]. These and other studies also reveal recruitment of posterior cortical structures including the angular gyrus [30], temporoparietal junction [33], posterior cingulate [30], and subcortical structures including the amygdala [34]. Complimenting the results of activation studies using fMRI, there is evidence that either the temporary disruption of [35], or damage to [36,37], these structures can alter moral reasoning.

### The current study

The present study sought to address four hypotheses that were motivated by our previous work using ERPs to examine the neural correlates of decision making related to information security, and the broader literature related to the role of self-control and moral belief in violations of information security:

Hypothesis 1: ERP activity elicited during the decision prompt should reveal a transient modulation of the ERPs over the posterior region beginning around 200 ms after stimulus onset that reflects greater negativity for control trials than for minor or major violations. Following this early transient activity, the ERPs should reveal sustained differences in amplitude over the frontal-central region reflecting greater positivity for minor and major violations than for control trials between 500–2000 ms; and differences in slow wave activity between control trials and minor and major violations over the lateral frontal region between 500–2000 ms after onset of the prompt.

This hypothesis was motivated by two goals. First, we sought to replicate the findings of the Hu et al. [13] study by using a sample of undergraduate students and university staff that represent a broader distribution of interests than the male undergraduate business majors included in our previous research. Second, we also sought to determine whether the ISP developed in Hu et al. [13] would continue to capture the neural correlates of decision making related to information security when the strong deception manipulation used in that study was removed from the paradigm.

Hypothesis 2: Moral potency will be correlated with modulations of the ERPs that distinguish violation scenarios from control scenarios as described in Hypothesis 1. Based upon existing theories within moral philosophy and psychology, the correlation between moral potency and the ERP correlates of ethical decision making could be either positive or negative depending upon how moral potency contributes to decision making [38,39]. Specifically, under the “grace” hypothesis of moral reasoning one could expect to observe a negative correlation between moral potency and ERP amplitude (i.e., lower amplitude with greater moral potency). This pattern would be consistent with the idea that moral potency serves to protect individuals from making unethical decision by lessening the attractiveness of potential benefits or instilling within them an absolute sense of right and wrong. In contrast, under the “will” hypothesis of moral reasoning one could expect to observe a positive correlation between moral potency and ERP amplitude (i.e., higher amplitude with greater moral potency). This pattern would reflect the idea that moral potency serves to potentiate controlled deliberative processes that allow one to resist the temptation inherent in the benefit associated with making an unethical decision [29].

Hypothesis 3: The amplitude of the ERPs that distinguish violations from control trials should be reduced in individuals with lower self-control relative to those with higher self-control; and the association between self-control and the ERP correlates of decision making related to information security will vary across the six subscales of the self-control questionnaire.

This hypothesis is motivated by the finding that subcomponents of self-control may be differentially related to the frequency of hacking behavior, and that this effect may interact with one’s motivation for the unethical behavior (i.e., whether it is exploratory or exploitative [15]). Based upon this evidence, one might expect the ERPs to be more strongly related to emotion regulation (tempe) and risk-taking than to impulsivity, self-centeredness, a preference for simple task, or level of physical activity.

Hypothesis 4: The direction of the correlation between self-control and moral potency, as related to the ERP correlates of decision making will be in the opposite direction This hypothesis is guided by the findings of previous research demonstrating that self-control and moral beliefs may have opposite effects on either the intention to violate information security policies [6] or hacking behavior related to exploratory or exploitative motivation [15].

## Materials and methods

### Ethics statement

This study was approved by the Institutional Review Board of DePauw University as an expedited study. Written informed consent was obtained. IRB approval: “As a member of the IRB, I received your proposal on Information Security and Decision Making for review on 6/9/16. I concur that the project fits the criteria for Category I review. Thank you for submitting such a thorough and thoughtful proposal, which has granted me an opportunity to write the following sentence in an IRB response. The project is approved as submitted. You and Emily may begin to collect data at your convenience.”

### Participants

Forty-eight individuals participated in the study and represented a mix of students and professional staff from a private liberal arts university in the midwest. The data for six individuals were not included in the analyses due to poor quality of the EEG data; the demographic and individual difference data for one additional individual were lost due to an internet error, so the ERP data for this individual were not included in the analyses. For the 41 participants included in the analyses, the average years of age was M = 19.59, SD = 2.61. The sample was 68% female, 75% White, 15% Asian, and 10% Hispanic or Latino(a).

### Materials

#### Information security paradigm

The information security paradigm was nearly identical to that used in Hu et al. [13] and the full set of materials is available online (https://osf.io/fqz37). Some minor changes were made to the priming information that described Josh and the text of the five practice and 45 test scenarios, these served to correct typographical errors that were present in the original materials or to add clarity to the prose. There were 15 instances of each type of scenario. The length of the scenarios ranged from 94–209 characters, and the length of prompts ranged from 19–68 characters. Examples of the three types of scenarios are presented in Table 1. The 15 control scenarios involved routine decisions in daily life that did not involve information security or an ethical component (scenarios M = 150, SD = 27 characters, prompts M = 40, SD = 8 characters). The 15 minor violation scenarios were related to various aspects of information security and could have moderate negative consequences were the action to be committed and the person caught by the organization (scenarios M = 151, SD = 16 characters, prompts M = 55, SD = 6 characters). The 15 major violation scenarios were also related to various aspects of information security and could have significant consequences were the action to be taken and the person caught by the organization (scenarios M = 170, SD = 22 characters, prompts M = 55, SD = 12 characters). During the task, the 45 scenarios were presented in a quasi-random order with the constraint that no more than three scenarios of the same type could appear consecutively.

**Table 1:** Examples of control, minor violation, and major violation scenarios and prompts.

Trial type	Scenario	Prompt
Control	Josh’s boss Christine asks Josh to spend a day helping her on a project she needs to complete by Friday.	Should Josh help Christine on her project?
Minor	Josh’s best friend Eric, who works for a competitor, wanted to know whether a new product under development has certain features.	Should Josh access the secure server and find the data?
Major	Josh’s mentor Mary was laid off due to downsizing. Josh is very upset about this and considering doing something for revenge.	Should Josh delete crucial computer files to vent his anger?

**Fig 1:**
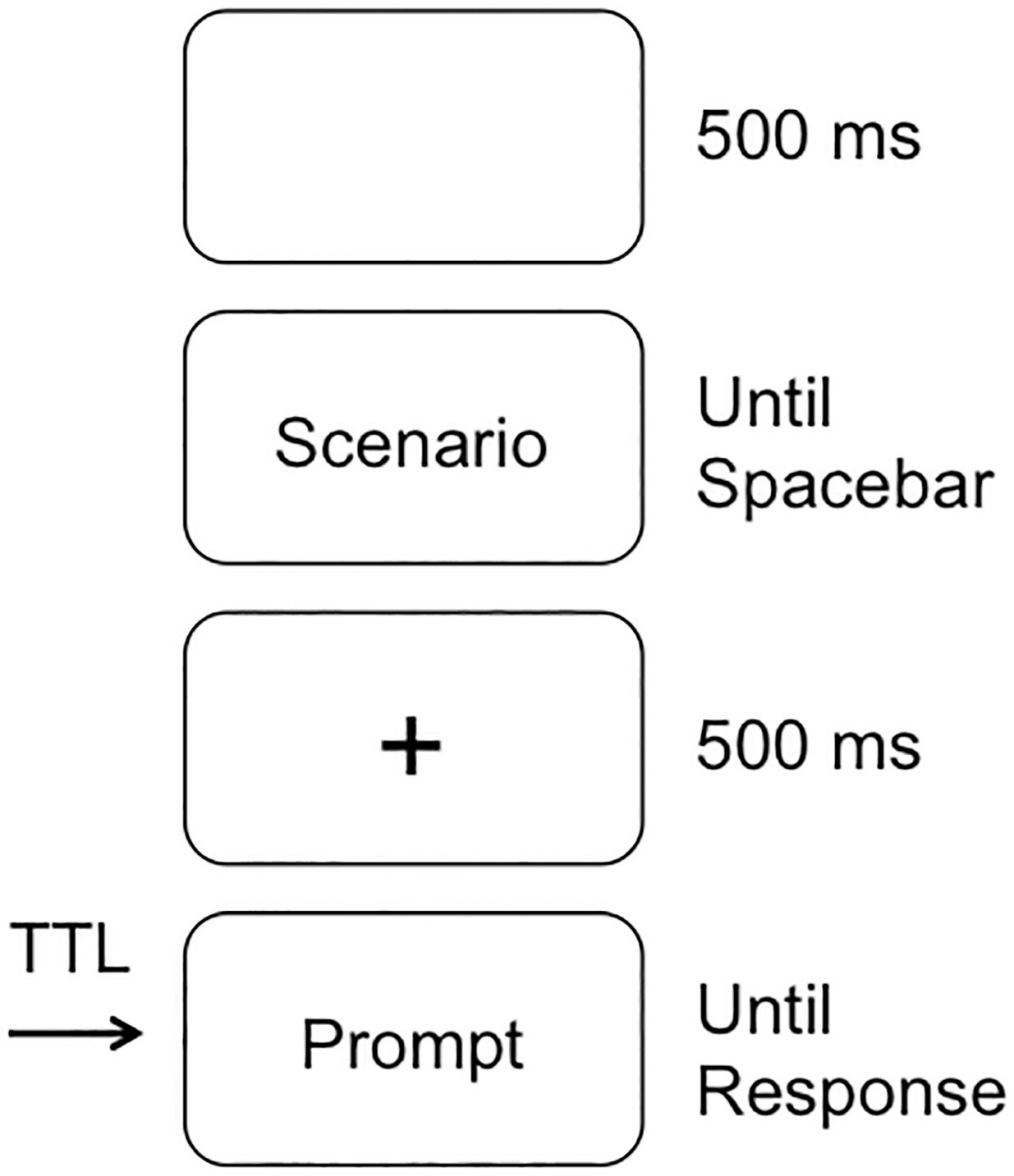
Sequence of events within a trial for the information security paradigm. The TTL pulse was aligned with onset of the Prompt, so that the response time and the ERP data are locked to onset of the prompt.

For the information security paradigm, the background information described Josh as a skilled IT professional with broad access to digital assests within his company; this information also indicated that the company had strict policies against unathorized use of assets, and that Josh was under time pressure for some projects in additional to financial strain. In responding to the scenarios, participants were asked to “imaging that you are Josh”. For each trial, a blank screen was presented for 500 milliseconds followed by the scenario that remained on the screen until the participant pressed the space bar to advance to the prompt (Fig 1). The scenario was followed by a fixation cross in the center of the screen that was presented for 500 milliseconds and followed by onset of the prompt. The prompt remained on the screen until a response was made and then the program advanced to the next trial. Participants were asked to response by pressing one of four keys on a USB keyboard (C-left middle finger, V-left index finger, B-right index finger, N-right middle finger) that were associated with four responses (No, Likely No, Likely Yes, Yes). A TTL trigger was delivered to the amplifier via a parallel port, that was aligned with the onset of the prompt.

#### Self-control scale

The self-control scale was identical to that used by Hu et al. [13]. The scale included 24 items that represented six constructs measured with four items each (Impulsivity, Risk taking, Self centered, Simple task, Physical activity, Tempe). Participants used a 7-point scale (1-Strongly disagree, 4-Neutral, 7-Strongly agree) to respond to the 24 items.

#### Moral potency scale

This scale was developed by Hannah and Avolio [20] and is designed to measure three aspects of the general construct moral potency (i.e., moral courage, moral ownership, moral efficacy). The scale includes 12 items and individuals responded using a 5-point scale (1-Strongly disagree, 2-Disagree, 3-Neither agree/Disagree, 4-Agree, 5-Strongly agree) with higher scores presenting greater moral potency.

#### Short handedness scale [40]

This scale included 10 items and was designed to measure an individual’s tendency to prefer the left or right hand when performing various simple tasks (e.g., writing, sweeping with a broom). Individuals responded by placing two plus signs in the column marked right or left if the activity was always performed with one or the other hand or distributed the plus signs between the left and right hand options is a specific hand was not preferred.

### Procedure

After arriving at the laboratory for the testing session, individuals received a brief overview of the study that described the EEG recording procedure and the individual difference and experimental tasks to be completed during the study. If the individual agreed to participate, signed informed consent was obtained. Then the participants head was measured to determine the appropriate size of the actiCAP. Individuals then completed the demographic and individual difference measures while the researchers prepared the actiCAP for application. After the questionnaires were completed, individuals were fitted with the actiCAP and then completed the information security paradigm followed by the two doors gambling task for a parallel study. After completion of the two computer tasks, the actiCAP was removed and individuals were debriefed and compensated for their participation. Compensation was either $20 ($10 for participation and $10 winning for the gambling task) for staff members, or research credits and $10 (representing winnings from the gambling task) for students.

### EEG recording and analysis

The EEG data were recorded with a 32 channel actiCAP and actiCHamp active Ag/AgCl electrode system using the Brain Vision Recorder software (Brain Vision, LLC). The EEG was sampled at 500 Hz, from DC-150 Hz with a 24 bit digitizer. Thirty electrodes were distributed across the scalp in the standard Brain Vision configuration (two inferior ocular electrodes replaced electrodes CP1-CP2), two electrodes were placed below the eyes to monitor blinks and vertical eye movements; and the ground electrode was placed at electrode Fpz. During recording the electrodes were referenced to electrode Cz, and electrode impedances were below 20 k**Ω**. The EEG was re-referenced to the average reference following the correction of ocular artifacts (i.e., blinks and saccades) using ICA. Triggers were delivered to the amplifier via a parallel port and were synchronized to the onset of the prompt.

Data processing was done using EEGLAB [41] and ERPLAB [42]. First a .1–30 Hz bandpass IIR filter (2^nd^ order, half amplitude -6 dB, half power -3 dB) was applied to the continuous EEG. Then an ICA was run to identify artifacts associated with blinks, and vertical and horizontal saccades. Visual inspection of the IC topography and time course (aligned to the raw EEG) was used to identify components reflecting ocular artifacts. Following the correction of ocular artifacts, up to two bad channels were corrected as needed using linear interpolation, the average reference was applied to the EEG data, and activity at electrode Cz was reinstated into the dataset. The ERPs were epoched from -200 to 2000 ms around the TTL pulse that was aligned to onset of the prompt, and remaining artifacts were rejected using a +/- 100 **μ**V absolute voltage threshold. Artifact free trials were averaged for control trials (M = 14.49, SD = .90), minor violations (M = 14.51, SD = .81) and major violations (M = 14.61, SD = .80).

Task Partial Least Squares (PLS) Analysis [43] was used to examine mean differences in ERP amplitude between the three task conditions. This allowed us to consider the data for all conditions, time points, and electrodes within a single analysis. This method also allows us to refrain from making decisions, which may be somewhat arbitrary, regarding the electrodes and time points to include in the analysis. The TaskPLS Analyses included data from 0–2000 ms after stimulus onset at 30 electrodes (i.e., all but the two ocular channels). The analyses were conducted with the ERP module of the PLSGUI (http://www.rotman-baycrest.on.ca) run under Matlab R2013b. Five hundred permutations and bootstrap samples were used to evaluate the significance of the latent variables and the stability of the electrode saliences. Time ranges reported in the Results section represent temporal epoch where the bootstrap ratio exceeded 2.0 that approximates p = .05.

Behavioral Partial Least Squares (PLS) analysis was used to examine brain-behavior relationships between the ERP data and the individual difference measures related to moral potency and self-control. This technique allows one to examine the correlation between one or more behavioral measures and the full time-course and topography of the ERP data. This represents a particularly attractive tool given that the effects of decision making on the ERPs are distributed in both space and time. For the BehavioralPLS analyses, a difference wave was calculated that represented the average ERPs for minor and major violation scenarios minus the average ERP for control scenarios. The PLS analyses included the difference wave ERPs for 0–2000 ms after onset of the prompt at 30 electrodes. Bootstrap resampling (500 iterations) was used to estimate the 95% confidence around the brain-behavior correlations. The scatterplots for an initial set of PLS analyses revealed one participant that was a multivariate outlier reflected by an extreme scalp score, therefore, this individual was removed from the analyses reported in the results section. In order to visualize the timing and distribution of the brain-behavior correlations, grand-averaged differences waves (minor and major violations minus control scenarios) using a median split for the relevant behavioral or individual different variable are presented in the Results section.

## Results

### Choice and response time data

The response choice data revealed a significant main effect of scenario, F(2,80) = 185.80, p < .001, d = 4.15, reflecting a decrease in the likelihood of responding yes from the control scenarios (M = 2.90, SD = .32) to the minor violation scenarios (M = 1.74, SD = .41, t(40) = 14.52, p < .001, d = 2.27) and from the minor violation scenarios to the major violation scenarios (M = 1.66, SD = .43, t(40) = 2.41, p = .02, d = .38). These findings are consistent with previous research [12] and indicate that individuals are in general less likely to endorse the unethical behaviors than the neutral behaviors, and are also sensitive to the severity of the ethical violation.

The response time data, measured in seconds, revealed a significant main effect of scenario, F(2,80) = 9.58, p < .001, d = .98, reflecting similar response time for control scenarios (M = 2.43, SD = .99) and major violation scenarios (M = 2.44, SD = 1.34, t(40) = .05, p = .96, d = .01), and longer response times for minor violation scenarios (M = 2.78, SD = 1.10) than for either control scenarios, t(40) = 4.05, p < .001, d = .63, or major violation scenarios, t(40) = 3.66, p < .001, d = .57. These findings are also consistent with previous research [13], and may reflect longer deliberation for committing the minor violations weighted against the potential benefits to be gained.

The correlations for the response choice and response time data for the three types of scenarios are reported in Table 2. The correlation between response choice was large and significant for minor and major violations, while response choice for control scenarios was not significantly correlated with response choice for either type of violation. These findings reveal both convergent validity (i.e., robust correlation between response choice for the violations) and discriminant validity (i.e., low correlations between choices for ethical and control scenarios) for the response choice data in the information security paradigm. The correlations between response time for the three types of scenarios were all high and significant, indicating that response time may be a reliable construct in the information security task, and that similar processes likely contribute to decision latency across the three types of scenarios.

**Table 2:** Pearson correlations between choice and response time for control, minor violation, and major violation scenarios.

	Choice			Response time		
	Control	Minor	Major	Control	Minor	Major
Choice Control	-					
Choice Minor	0.05	-				
Choice Major	-0.12	0.85*	-			
RT Control	-0.30	-0.16	-0.10	-		
RT Minor	-0.25	0.15	0.15	0.86*	-	
RT Major	-0.34	0.03	0.08	0.91*	0.89*	-

Note:

* is p < .001

### Individual difference data

The reliability for the full 12-item Moral Potency Scale was acceptable for a research instrument, Cronbach’s **α** = .72, 95%CI[.58-.83]. The reliability of the three subscales was below **α** = .70 (Moral Courage, Cronbach’s **α** = .67, 95%CI[.46-.81], Moral Efficacy, Cronbach’s **α** = .62, 95%CI[.41-.78], Moral Ownership subscale, Cronbach’s **α** = .35, 95%CI[-.09-.63]). Given these results, we decided to treat moral potency as a single composite measure averaged across the 12 items in the brain-behavior analyses.

The reliabilities for the six subscales of the self-control scale were all acceptable for experimental measures (Table 3) and were greatest for the risk-taking and physical activity subscales. Given these findings, examining brain-behavior associations at the subscale level for self-control is justified.

**Table 3:** Cronbach’s α for the six subscales of the self control scale.

	Impulse	Risk Taking	Self Centered	Simple Task	Physical Activity	Tempe
Cronbach’s **α**	.70	.88	.71	.76	.87	.75
95% CI	.51-.82	.81-.93	.53-.83	.62-.86	.80-.93	.59-.85

### ERP data

**Fig 2:**
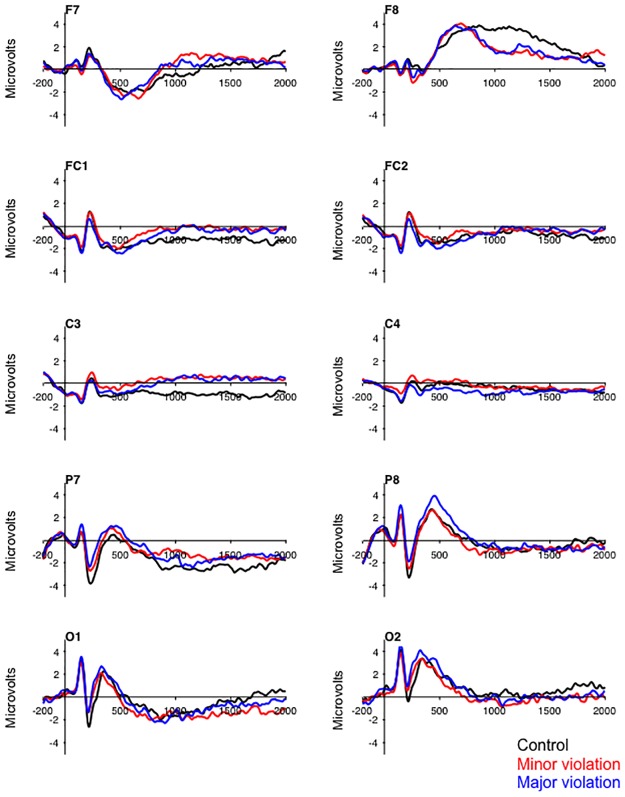
Grand-averaged ERPs for 41 subjects at 10 electrodes demonstrating the timing of differences in ERP amplitude between the control scenarios and the minor and major scenarios.

Fig 2 includes a subset of the electrodes portraying modulations of the ERPs that distinguish minor and major violation scenarios from control scenarios. These data reveal modulations of the ERPs between 200–2000 ms after onset of the prompt that differ for the control scenarios and the minor and major violation scenarios.

**Fig 3:**
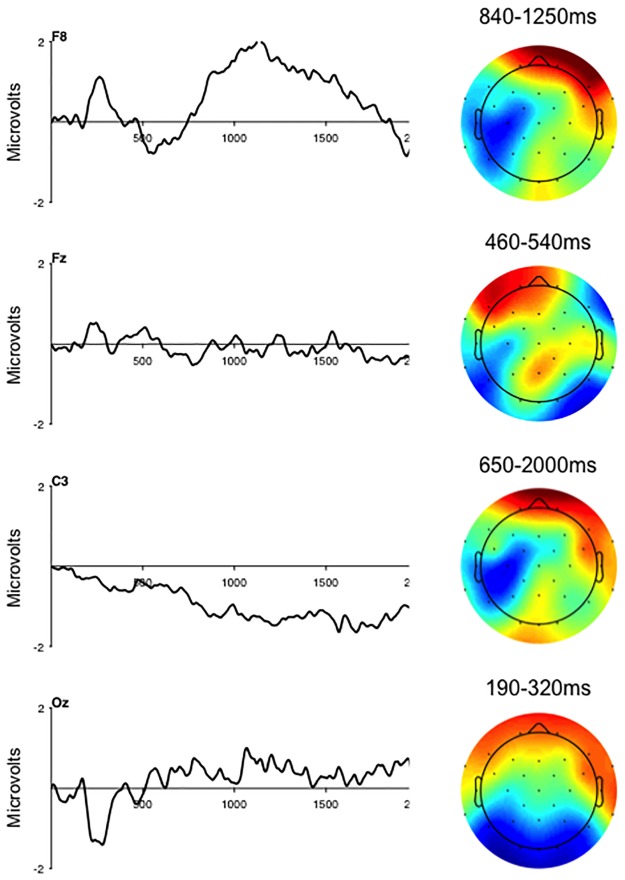
ERP saliences from the TaskPLS analysis at four electrodes and topography maps demonstrating the spatial distribution of the electrode saliences for the first latent variable that contrasted minor and major violation scenarios with control scenarios. The times represent point where the bootstrap ratio exceeded 2.0. Topographic maps represent the mean voltage over the time window indicated and are to maximize the voltage gradient, ranging from +/-1.0–2.0 microvolts.

The first latent variable from the TaskPLS analysis provides support for Hypothesis 1, and reveals a general effect of ethical decision making on the ERPs that was insensitive to the severity of the violations. The first latent variable contrasted the control scenarios (contrast weight = .80) with the minor (contrast weight = -.53) and major (contrast weight = -.28) violation scenarios and accounted for 72.65% of the crossblock covariance (p = .002). The electrode saliences for this latent variable revealed a transient modulation of the posterior N2 component between 190–320 ms after onset of the prompt (Fig 3, electrode Oz). This effect on the posterior N2 was coupled with transient modulations over the medial frontal region (electrode Fz) and over the right hemisphere extending from the frontal-polar region to the temporal-parietal region (electrode F8). The early transient modulations were followed by transient modulations over the medial frontal region between 460–540 ms, and over the right lateral frontal region between 840–1250 ms (electrode F8). Finally this contrast was associated with slow wave activity that extended from the frontal-central to the parietal region over the left hemisphere (electrode C3), and from the frontal-polar to the lateral frontal region over the right hemisphere (650–2000 ms). The pattern of electrode saliences for the first latent variable is consistent with previous results [13] in demonstrating that ethical decision making as related to information security is associated with sustained activity over the frontal and central regions of the scalp.

The second latent variable contrasted minor violation scenarios (contrast weight = .62) with major violation scenarios (contrast weight = -.77) with a small contribution from control scenarios (contrast weight = .15). This latent variable accounted for 27.35% of the crossblock covariance, but not differ from chance (p = .77). Therefore, in contrast to the findings of Hu et al. [13], the current study does not reveal ERP activity that reliably distinguishes between minor and major violations (i.e., that is sensitive to the severity of the violation).

### Brain-behavior relationships

**Fig 4:**
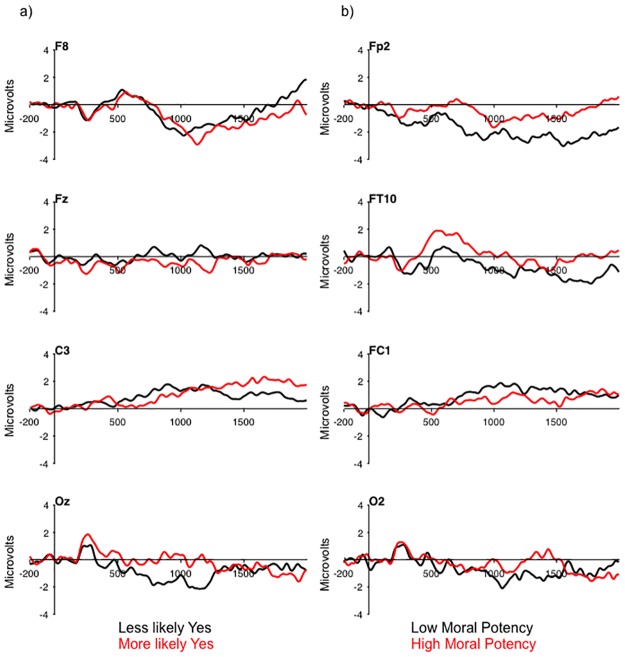
a) Grand-averaged difference waves (violation minus control) collapsed across minor and major violation scenarios reflecting a median split for choice on minor and major violations. b) Grand-averaged difference waves (violation minus control) reflecting a median split for moral potency.

Before examining the brain-behavior relationships tied to Hypotheses 2–4, we explored the relationship between the choice data for minor and major violation scenarios and the ERP data representing the difference in amplitude between the average of minor or major violation scenarios and the control scenarios in a BehavioralPLS analysis. This analysis revealed negative correlations between the ERP scalp scores and choice for minor violation scenarios (r = -.71, 95%CI [-.65,-.84]) and major violation scenarios (r = -.63, 95%CI[-.57,.83]). These correlations reflected greater ERP amplitude for those individuals with higher scores (i.e., more likely to choose Yes) relative to those individuals with lower scores over the occipital and medial frontals at the time of the N2/P2, and lateral frontal and central regions of the scalp from around 1000–2000 ms after onset of the prompt (Fig 4a). These findings demonstrate that there is a reasonably strong relationship between choice behavior in the information security paradigm and neural activity that distinguishes violation scenarios from control scenarios. Furthermore, these data indicate that the neural system supporting decision making in the information security paradigm is more strongly recruited in those individuals who are more likely to endorse an unethical action within the task.

The second BehavioralPLS analysis addressed Hypothesis 2 by examining the relationship between moral potency and the violation minus control scenario difference wave. Supporting Hypothesis 2, this analysis revealed a positive correlation between moral potency and ERP amplitude (r = .77, 95%CI[.71,.87]). This correlation represented greater ERP amplitude over the medial frontal, lateral frontal, and frontal-central regions for individuals with lower moral potency relative to those individuals with higher moral potency (Fig 4b). In contrast, the amplitude of the posterior N2 was not sensitive to individual differences in moral potency (Fig 4b electrode O2). This finding may indicate that individuals who possess higher moral potency give less consideration to potential violations of information security, or that higher moral potency may temper the attractiveness of unethical decisions.

**Fig 5:**
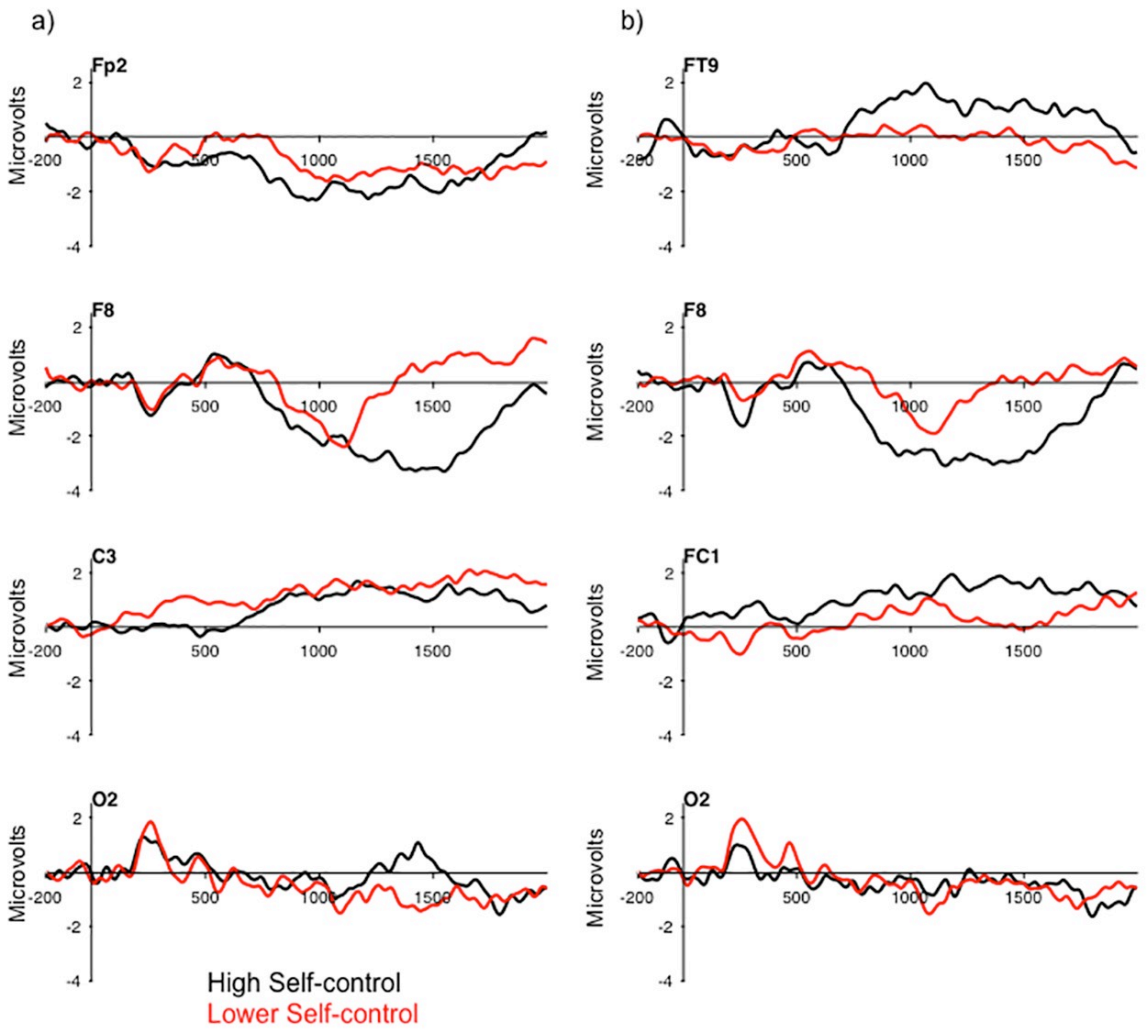
a) Grand-averaged difference waves (violation minus control) reflecting a median split for average of self-centered, simple task, and physical activity. b) Grand-averaged difference waves (violation minus control) reflecting a median split for average of impulsivity, risk-taking, and tempe.

The third BehavioralPLS analysis addressed Hypothesis 3 by examining the relationship between the six subscales of self-control and the ERP data. This analysis provides support for Hypothesis 3, and revealed two patterns of brain-behavior correlations representing ERP activity that was broadly distributed across the scalp (Fig 5a and 5b). Consistent with the analysis of moral potency, these brain-behavior correlations were related to the ERPs over the occipital, and lateral frontal regions of the scalp. The first reflected negative correlations between ERP activity, and the self-centered and simple tasks subscales, and a positive correlation between ERP activity and the physical activity subscale (Table 4). This pattern reflected lower ERP amplitude for individuals with higher scores (i.e., those that possessed lower control) on the three subscales (i.e., were more self-centered, had a preference for simple tasks, and preferred physical over mental activity) than for those individuals that scored lower in the three subscales. The second represented negative correlations between ERP amplitude and impulsivity, risk-taking and tempe (Table 4). This pattern also reflected lower ERP amplitude for individuals that scored higher on these three scales (i.e., were more impulsive, more risk-seeking, and exhibited poorer emotion regulation). Together, the results of this analysis indicate that there may be two higher order components of self-control that are both related to the under recruitment of the neural system underpinning ethical decision making related to information security.

**Table 4:** Brain-behavior correlations with 95% confidence intervals for the six subscales of self-control and ERP activity.

	Impulsivity	Risk-taking	Self-centered	Simple tasks	Physical activity	Temper
LV1	-.14 [-.41,.17]	.04 [-.24,.37]	-.43 [-.25,-.68]	-.32 [-.11,-.65]	.64 [.49,.83]	-.06 [-.43,.29]
LV2	-.49 [-.41,-.73]	-.47 [-.43,-.75	-.21 [-.54,.15]	.29 [-.21,.55]	-.06 [-.57,.29]	-.31 [-.03,-.60]

A final set of BehavioralPLS analyses addressed Hypothesis 4, and examined the relationship between moral potency, the two components of self-control, and the ERP data. One analysis included moral potency, self-centered, simple tasks, and physical activity; and the other included moral potency, impulsivity, risk-taking, and tempe. In support of Hypothesis 4, the correlations between moral potency and self-control were either in the opposite direction (e.g., positive for moral potency and negative for self-control) or moral potency and self-control were related to different latent variables (Table 5). These findings may indicate that moral potency and various aspects of self-control have either opposing or indepdent effects on the recruitment of the neural system that underpins decision making related to information security.

**Table 5:** Brain-behavior correlations and 95% confidence intervals for moral potency, the two components of self-control, and ERP activity.

	Moral potency	Self centered	Simple tasks	Physical activity
LV1	.02 [-30,.39]	-.43 [-.27,-.70]	-.35 [-.18,-.66]	.63 [.48,.82]
LV2	.63 [.53,.81]	-.14 [-.50,.15]	-.43 [-.14,-.70]	-.40 [-.003,-.68]
	Moral potency	Impulsivity	Risk-taking	Temper
LV1	.14 [-.13,.54]	-.56 [-.44,-.75]	-.54 [-.45,-.76]	-.44 [-.20,-.66]
LV2	-.71 [-.63,-.86]	-.24 [-.50,.09]	-.12 [-.40,.21]	.14 [-.14,.49]

## Discussion

This study was designed to provide a replication and extension of previous research examining the neural correlates of decision making related to violations of information security policy. The study focused on four hypotheses that were either motivated by the previous research on self-control and information security decision making or the larger empirical literature related to self-control, moral belief, and decision making. Analyses of the behavioral and ERP data, and an examination of brain-behavior relationships, provide full or partial support for each of the four hypotheses. Given this, the current data may serve to build upon and extends our understanding of the influence of individual difference in self-control and moral belief on the neural architecture underpinning decision making in the context of information security.

Hypothesis 1 focused on replicating the results of previous research [13] in a sample with more diverse academic and professional interests, and when the strong deception manipulation was removed from the research paradigm. The results of the TaskPLS analysis provided partial support for Hypothesis 1. Consistent with the previous findings [13], the contrast of minor and major violation scenarios with control scenarios revealed reliable differences in ERP activity beginning around 200 ms after onset of the prompt and continuing until at least 2000 ms. The early modulations of the ERPs were localized over the occipital, and medial and lateral frontal regions, while the later modulations were localized over the anterior and lateral frontal region, and the central and temporal regions. Diverging from previous findings, the current data did not reveal reliable differences in ERP amplitude between minor and major violations of information security. These findings demonstrate that deception is not required to reliably engage the neural network underpinning decision making in the information security paradigm; and are important as they reveals that individuals will engage in the task when performance is untethered from expectations grounded in the reward structure of the paradigm.

The effect of scenario type on the posterior N2 and frontal P2 represented a decrease in the amplitude of the components for information security violation scenarios relative to control scenarios. Given the visual and semantic complexity of the prompts, and the fact that response time was relatively slow across the three types of scenarios (i.e., 2000 ms), it seems unlikely that the effects observed for these components arise from the properties of the prompts. This then leads one to wonder what psychological processing might give rise to these effects. The posterior N2 and frontal P2 are both known to be sensitive to the allocation of attention to stimuli in the visual environment [44]. The amplitude of these components is typically greater when attention is directed to a goal-relevant stimulus attribute and when more attentional resources are available for stimulus processing [44]. For instance, the amplitude of the posterior N2 and frontal P2 is attenuated for visual stimuli when individuals are also trying to maintain two or more items in working memory [45]. In the context of this literature, the attenuation of the posterior N2 and frontal P2 for information security violations may reflect the inward allocation of attentional resources directed toward weighing the potential benefits and risks of the unethical acts represented in the scenarios that results in the attenuation of visual processes devoted to encoding of the decision prompt.

The medial and lateral frontal, and right central modulations of the ERPs that distinguished minor and major violation scenarios from control scenarios are also consistent with previous findings [13]. These data also converge with the broader moral reasoning literature revealing the contribution of structures within the medial and lateral frontal cortex, and the temporal and parietal lobes to ethical decision making [29]. The neuro-cognitive processes represented by the sustained modulations of the ERPs remain unclear based upon the available evidence. One possibility is that the information security paradigm elicits activity in elements of the mentalizing or prospection network [46] related to weighing the potential costs and benefits embodied in the scenarios in a way that differs from the recruitment of this network when considering the control scenarios. A second possibility is that the medial and lateral frontal activity reflects processing that serves to resolve conflict that arises between the potential gains and penalties represented in the scenarios [47]. The medial and lateral frontal ERP activity might reflect the recruitment of structures involved in the evaluation of the utility associated with the potential gain or loss related to accepting the unethical actions represented in the minor and major violation scenarios [47]. Importantly, these are not mutually exclusive accounts and future research could serve to untangle the contributions of these different processes to the ERPs related to decision making in the information security paradigm.

Hypothesis 2 examined the relationship between moral potency and ERP activity that distinguished violations of information security from control scenarios. An examination of the psychometric properties of the moral potency scale revealed that the full scale provided a reliable construct, while the three subscales did not. The BehavioralPLS analysis related to this hypothesis revealed a strong correlation between moral potency and ERP amplitude. This correlation represented greater ERP activity for individuals low in moral potency relative to those high in moral potency over the medial, lateral, and anterior frontal regions of the scalp, but not over the occipital region at the time of the N2. This finding provides support for the *grace* hypothesis, rather than the *will* hypothesis [30,38], and may indicate that higher moral potency serves to insulate individuals from the temptation presented by the potential for ill-gotten gains represented by the violations of information security. The correlation between moral potency and the ERPs related to ethical decision making in the context of information security extends upon the extant literature in this domain that has focused on moral beliefs specifically related to information security [15,26], demonstrating a more general effect of the strength of individual moral beliefs on neural recruitment when contemplating an unethical behavior.

Hypothesis 3 examined the relationship between the ERPs related to information security decision making and the six subscales of the self-control scale. The BehavioralPLS analysis revealed two latent variables that distinguished the six subscales. One representing self-concern, a preference for simple tasks, and a preference for physical activity; and one representing impulsivity, risk-taking, and tempe. Higher scores on the subscales were associated with reduced ERP amplitude, which may indicate that individuals with lower self-control are less likely to consider or deliberate over the costs and benefits represented in the ethical violations scenarios, and thus being more likely to base their decisions upon more automatic non-deliberative processes [29,39]. This finding nicely converges with the broader decision making literature demonstrating that cooperation and moral behavior often require the engagement of controlled deliberative processes [48,49].

In Hypothesis 4 we proposed that the direction of the correlation between ERP activity and moral potency and self-control would be in the opposite direction. The analyses provided partial support for this hypotheses, and revealed two patterns of association. For one of the four latent variables, the correlation was positive for moral potency and negative for two of the three subscales of self-control. This pattern is consistent with the idea that moral potency and self-control may have opposing effects on the neural correlates of decision making [6, 15]. For the other three latent variables, the association between the ERP activity and the individual difference measures was reliable for either moral potency or self-control. Thus, taken together it would seem that across the four latent variables the most consistent description of the pattern of correlations is that moral potency and self-control have independent, rather than opposing, effects on neural activity related to decision making related to information security. However, this finding diverges from some findings in criminology literature wherein moral belief moderates the influence of self-control in decision making that involves unethical behavior such as crime [48,49], revealing that the effect of self-control is only significant in the individuals with lower morality. Multiple factors could have contributed to this discrepancy, such as differences in measurement scales for moral potency and self-control and the dependent variable. But it nonetheless remains an interesting research subject for future study.

One of the motivations for exploring the neural correlates of information security decision making is to identify the processes that may contribute to policy violations arising from insider threat [13]. Such knowledge may provide insight into the factors that contribute to violations of information security policy and the limited success of deterrence programs [6]. Deterrence programs are often built upon a set of rules or regulations that are designed to insure the protection of an organization’s digital assets. Our data may indicate that the failure of individuals with low self-control to engage deliberative processes that facilitate the application of rules or regulations during decision making could contribute to violations of information security policy. The current data and those of other investigators, also lead to the idea that high moral belief or identity may serve to insulate individuals from the effect of low self-control related to either individual differences or temporary depletion [16,17], and that priming moral thinking can have a significant effect on the outcome of decision making [22,23]. Together this literature may serve to support the refinement of deterrence programs thereby reducing instances of insider threat.

### Limitations

There are some limitations to the current study that are worthy of consideration. First, one could question the ecological validity of the paradigm given the large number of security violations that individuals are faced with in the task. The information security paradigm was modeled after a method used in behavioral studies of information security wherein individuals are only posed with a small number of examples (e.g., three). In our previous work [13] and in the current dataset, we observed that individual differences in self-control, that are known to be predictors of violations of information security in natural settings [15,48], were consistently related to variation in neural recruitment providing some indication that our implementation of the information security paradigm may capture decision making processes that are relavant to decision making in more naturalistic situations. Second, the current sample is primarily female, and there is evidence from the NeuroIS literature that neural recuitment related to decision making in the context of trust may differ in males and females [50]. Our previous research examining the neural correlates of information security included only males, so a logical next step for this program of research would be to conduct a study with a gender balenced sample that would permit the direct comparison of gender differences in neural recruitment in the task. Third, we did not assess participants experience with violations of information security or knowledge of information security policies and practices. This would clearly be an interesting question to consider in future research as it could provide insight into both the ecological validity of the information security paradigm, and the impact or effectiveness of training in information security on decision making. Fourth, the ERPs related to decision making were measured over a relatively long epoch (i.e., 2 seconds) and the task includes only three conditions. This makes it difficult to clearly map cognitive processes to the ERPs that distinguished control scenarios from ethical violation scenarios. The prompts for each type of scenario require encoding a visual stimulus that represents a sentence, making a decision related to an action to be taken, and selecting a response. So these fundamental cognitive processes would not seem to capture the differences observed across the three task conditions. Therefore, one goal of future research could be to further elucidate the cognitive processes that are reflected in both the transient and sustained ERP activity that distinguishes the various trials in the information security paradigm. Finally, differences in response choice could influence the ERPs as responses for the minor and major violations would primarily reflect use of the left hand given the means were below two, while responses for the control trials would reflect a greater number of right hand responses given that the mean was around three. The left central slow wave that distinguishes security violation scenarios from control scenarios does not reveal a reversal over the right central region as would be expected if this ERP activity reflected the preparation or execution of a motor response that differed between the control and violation scenarios. However, research that controls for differences in responses between control and violation trials, and yet continues to reveal ERP activity that distinguishes between these two types of trials, represents an important future step in this line of research.

### Conclusions

The findings of the current study provide full or partial support for the four hypotheses, and demonstrate that the information security paradigm is associated with the recruitment of a neural network that is not dependent upon the use of deception to motivate participants task performance. Higher moral potency may serve to decrease the attractiveness of information security violations. While low self-control may result in a decrease in the degree to which individuals engage deliberative processes when faced with choices to violate or not to violate information security policies. Perhaps the most interesting finding is that moral potency and self-control appear to have somewhat independent influences on neural activity in the information security paradigm, supporting the idea that both deliberative and non-deliberative processing are involved in decision making related to information security [38,39].
